# Performance of image-guided bone biopsies in malignant lesions: impact of PET/CT metabolic activity on the number of samples required

**DOI:** 10.1186/s13244-025-02130-2

**Published:** 2025-10-31

**Authors:** Mathieu Conjeaud, Rémi Grange, Vincent Habouzit, Claire Boutet, Michel Peoc’h, Pierre-Benoit Bonnefoy, Sylvain Grange

**Affiliations:** 1 Department of Radiology, University Hospital of Saint-Etienne, Saint-Etienne, France; 2 Department of Nuclear Medicine, University Hospital of Saint-Etienne, Saint-Etienne, France; 3 TAPE Research Unit EA 7423, Saint Etienne, France; 4 Department of Pathology, University Hospital of Saint-Etienne, Saint-Etienne, France; 5 Mines Saint-Etienne, INSERM, SAINBIOSE U1059, Saint-Etienne, France

**Keywords:** Bone biopsy, Number of samples, PECT/CT, Metabolic activity, Cancer

## Abstract

**Objective:**

The purpose of the present study is to determine whether or not lesion characteristics on PET/CT could reduce the number of samples required to achieve a diagnosis in image-guided bone biopsies (IGBB).

**Materials and methods:**

A retrospective review of 38 percutaneous IGBB performed at a single center. Biopsies have been performed from January 1st, 2020, to October 23rd, 2024. Inclusion criteria were patients with a PET/CT and a histopathologic report available. Specimens were collected, numbered, and independently analyzed in separate containers. PET/CT data, including SUV_max_, SUV_mean_, MTV, TLG, and morphological lesion characteristics, were correlated with biopsy outcomes and subjected to statistical analysis. Patients were classified by the number of samples needed for diagnosis: first (Group 1), second (Group 2), or third/subsequent (Group 3).

**Results:**

Thirty-four/38 (89%) involved spinal and pelvic locations (34/38; 89%). Breast cancer metastases were the most common diagnosis (21/38; 55%). Group 1 included 20 IGBB (52%), group 2 included 9 IGBB (24%), and group 3 included 9 IGBB (24%). No statistically significant difference was found between groups in metabolic characteristics and the number of samples needed for diagnostic purposes (*p* > 0.05). Subgroup analysis, including factors such as density or lesion size, didn’t find any significant differences between groups.

**Conclusion:**

The results suggest that high metabolic activity alone does not justify reducing the number of biopsy samples without compromising diagnostic performance. This supports the recommendation to obtain at least three samples and highlights the importance of selecting the safest biopsy site, regardless of metabolic activity.

**Critical relevance statement:**

This study critically assesses the role of FDG PET/CT metabolic parameters in predicting the diagnostic success of IGBB, providing new insights to improve target selection and biopsy planning in clinical radiology.

**Key Points:**

- This study assessed whether metabolic activity on FDG PET/CT influences the diagnostic yield of IGBB.
- High metabolic activity did not allow for reducing the number of samples without affecting diagnostic performance.
- At least three biopsy samples should be obtained, prioritizing safety over metabolic activity when selecting the biopsy site.

**Graphical Abstract:**

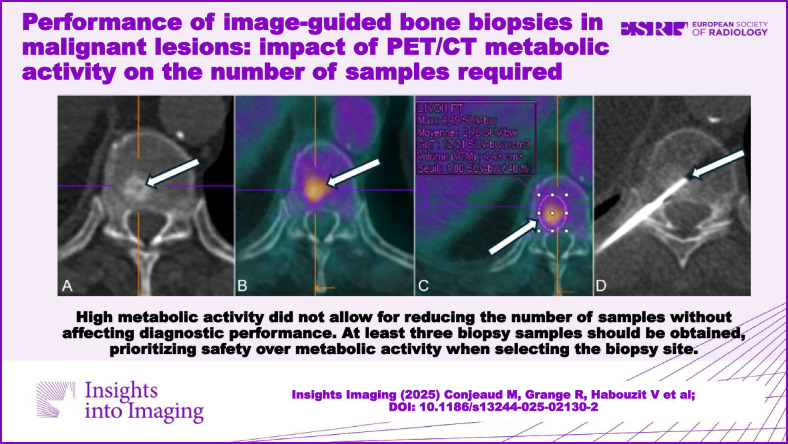

## Introduction

When a suspected malignant bone lesion is identified, obtaining a definitive diagnosis is essential. Image-guided bone biopsies (IGBB) are commonly performed as a first-line procedure, as they are less invasive than open surgical biopsy [1–5]. They are used to establish an initial diagnosis or as part of a staging workup in cases of known oncologic disease [6]. Moreover, IGBBs are increasingly valuable for detecting genetic mutations and guiding personalized therapies, including immunotherapy [1–3].

These procedures offer a high diagnostic yield, with a low complication rate. They can be performed in almost all anatomical locations and are often feasible on an outpatient basis [1, 7–13]. The literature reports that the diagnostic yield for IGBB procedures varies significantly, with studies showing rates from 69% to 98% [2, 11, 14–16]. Nevertheless, the literature is limited and does not provide clear recommendations on the optimal number of samples to obtain per biopsy procedure. Wu et al [17] observed that a plateau in diagnostic yield was reached after the third sample. Similarly, Wong [9], Droste et al [14], and Winkler et al [11] supported this observation. Moreover, the number of biopsy samples is a key factor influencing several procedural parameters, such as procedure duration, radiation exposure, pain, and type of anesthesia choice [12]. As the number of biopsy samples increases, so do all these parameters [4].

However, obtaining high-quality samples can be challenging, as lesion characteristics such as location, size, and density [2, 13, 18, 19] may present technical challenges [4, 6, 14–16, 18, 20]. Consequently, failing to optimize sample collection or selecting a suboptimal biopsy site can lead to diagnostic errors or inconclusive results, thereby increasing the need for repeat biopsies [21]. In clinical practice, biopsy procedures often require a trade-off between various factors, such as diagnostic performance, procedural safety, and patient comfort. The goal is to achieve the best possible balance, ensuring sufficient tissue sampling while minimizing risks, including radiation exposure, procedural duration, and patient discomfort. Optimizing biopsy techniques, particularly determining the minimum number of samples required to achieve optimal diagnostic performance, remains a key issue in interventional radiology. Moreover, positron emission tomography/computed tomography (PET/CT)-based guidance has been associated with enhanced diagnostic performance [14, 22–24]. FDG uptake is theoretically correlated with the proportion of active tumor cells [25], suggesting that targeting areas with high uptake could increase the likelihood of obtaining a diagnostic biopsy [20, 26–28]. Given that a significant number of patients undergo PET/CT as part of their clinical management [3, 29], the metabolic characteristics of biopsied lesions may improve procedural efficiency and reduce the number of required samples. This could also impact the selection of the biopsy site in cases of multiple suspicious lesions, potentially prioritizing a metabolically active but technically challenging lesion over a more accessible but less active one. To our knowledge, the influence of metabolism on biopsy samples needed has never been studied.

The primary objective was to evaluate whether performing bone biopsies in a more hypermetabolic area would result in a diagnosis with fewer samples. The second objective was to identify additional predictive factors for optimizing biopsies targeting suspicious bone lesions.

## Materials and methods

### Patients

**Fig. 1:**
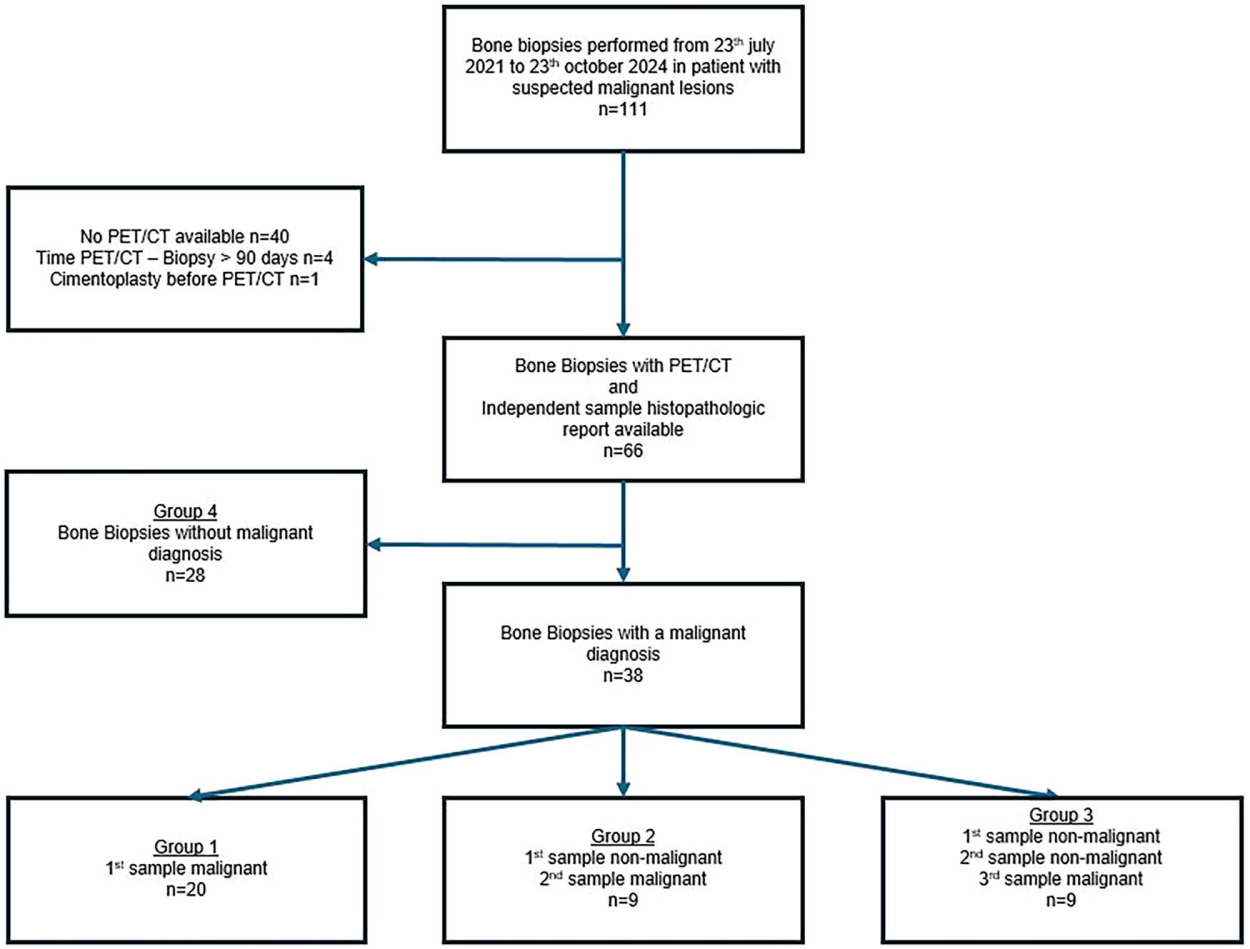
Flow chart of the study population

Biopsies were reviewed over an extended period from January 1st, 2020, to October 23rd, 2024, within the interventional radiology department at University Hospital of Saint-Etienne, France. Among 111 bone biopsies suspected of malignancy, 66 IGBB with a PET/CT and an independent sample histopathologic report were available. Of these, 28 had a non-malignant diagnosis and were not included in the statistical analysis. The Flow Chart is presented in Fig. 1. The data included patients’ demographics, biopsy technique, and imaging data, independent histopathology analysis, metabolic characteristics, and clinical and radiological follow-up. The hospital’s ethics committee approved this retrospective study (Terre d’Ethique, IRBN1412024/CHUSTE).

### Study design

A retrospective review of all IGBB was performed. Patients’ characteristics are presented in Table 1. The results of 66 percutaneous bone biopsies were analyzed, with 38 of these providing a pathological diagnosis. Only patients with 18F-FDG PET/CT were included; patients who had PET/CT with other tracers were not included in the analysis. Patients who underwent PET/CT more than 90 days before IGBB were excluded. When a patient underwent two biopsy procedures, only one was considered, because if one was pathological, the second was more likely to be pathological as well. In cases where both biopsy procedures were positive, only the first one was retained. If one procedure was positive and the other negative, only the positive one was considered. Two lesions were necrotic and were not included in the statistical analyses.

**Table 1:** Clinical and demographic data of patients with pathologic biopsy

Characteristic	All	Group 1	Group 2	Group 3	Group 4
Age (median; IQR)	69 (19)	68.5 (18.75)	72 (3)	62 (25)	67.5 (15.25)
Sex (female)	41/66 (62%)	17/20 (85%)	6/9 (67%)	6/9 (67%)	12/28 (43%)
Anesthesia modalities					
General	23/66 (35%)	6/20 (30%)	3/9 (33%)	3/9 (33%)	11/28 (39%)
Local	43/66 (65%)	14/20 (70%)	6/9 (67%)	6/9 (67%)	17/28 (61%)
Guidance					
Ultrasound	1/66 (1.5%)	0/20 (0%)	0/9 (0%)	1/9 (11%)	0/28 (0%)
Fluoroscopy	24/66 (36%)	9/20 (41%)	4/9 (44%)	3/9 (33%)	8/28 (29%)
CT	41/66 (62.5%)	11/20 (59%)	5/9 (56%)	5/9 (56%)	20/28 (71%)
Cancer					
History of cancer	51/66 (77%)	17/20 (85%)	6/9 (67%)	8/9 (89%)	20/28 (71%)
Previous or ongoing cancer treatment	13/66 (20%)	4/20 (20%)	2/9 (20%)	1/9 (11%)	6/28 (21%)

*IQR* interquartile range

### Procedure

Biopsies were performed to diagnose primary cancer, synchronous or metachronous bone metastases.

**Fig. 2:**
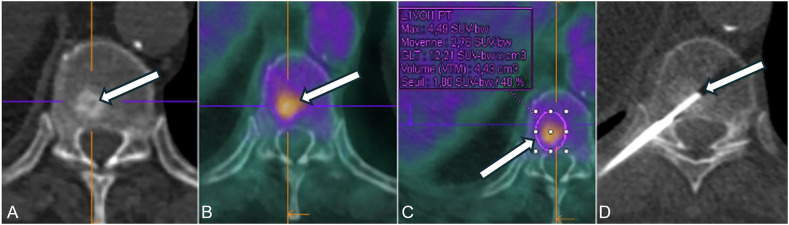
A 68-year-old patient with a history of bronchogenic carcinoma, surgically treated in 2023. The following imaging revealed a hypermetabolic sclerotic lesion at T10. **A** An uninjected CT presented in axial section corresponding to the vertebrae. **B** PET/CT with hypermetabolic vertebral lesion. **C** PET/CT with region of interest and metabolic parameters. **D** CT-guided core biopsy of vertebral lesion, with needle and trephine

Most biopsies (58/66) were performed by a senior musculoskeletal interventional radiologist with over 12 years of experience; the remaining 8 were performed by another radiologist with 6 years of experience. Bone biopsies were performed using coaxial techniques under ultrasound, CT, or real-time fluoroscopic guidance after CBCT acquisition (Fig. 2). When fluoroscopic guidance was chosen, a 3D real-time navigation software (*XperGuide*^®^, Philips Healthcare) planning software was used. When CT (SIEMENS Confidence^®^) guidance was chosen, a planning acquisition was initially conducted, followed by a series of short-range scan acquisitions after each needle adjustment. The method involved collecting and labeling containers separately, followed by independent analysis by pathologists. For needle entry and trajectory, a short and direct path was preferred, avoiding vascular, neural, or joint structures. Choice for imaging guidance depended on the radiologist's preference, the location of the lesion, and the availability of equipment. The use of local or general anesthesia was discussed during the consultation prior to the procedure, considering the patient’s preferences, anesthetic options, and whether additional procedures such as cementation were planned. Coaxial technique was used, including 11 and 13-gauge needles (T’AM KIT^®^, STRIM Health Care, or Arrow OnControl Powered Bone Lesion Biopsy System^®^, Teleflex). Local anesthesia (LA) to the skin and periosteum was administered. The trocar was then inserted into the bone to reach the lesion’s periphery, followed by the insertion of the trephine. Three specimens were collected as the standard in our institution. Biopsy specimens were visually assessed, placed in separate formalin containers, and sent for histopathological analysis.

### Lesion data

PET/CT scans were performed for all patients using an mCT Flow scanner (Siemens). Each PET/CT was retrospectively reviewed on a dedicated workstation (Syngo.via^®^, Siemens) to assess the morphological and metabolic characteristics of lesions that were biopsied. Morphological parameters such as lesion density, size, sclerotic or lytic components, and anatomical location were recorded. Lesion density was determined using a circular region of interest (ROI) placed on the axial slice showing the maximal lesion diameter. A lesion was considered sclerotic if more than 50% of its area exhibited higher density than the surrounding normal bone.

Metabolic parameters were also recorded for all lesions, including the standardized uptake values (SUV_max_, SUV_mean_), metabolic tumor volume (MTV) defined using a 40% SUVmax threshold, and total lesion glycolysis (TLG), calculated as SUV_mean_ × MTV. For each patient, SUV_mean_ was measured in reference regions (aortic arch and liver), and tumor-to-blood and tumor-to-liver uptake ratios were calculated.

### Histopathologic diagnosis

Once obtained, biopsy samples were sent to the pathology department. Samples were analyzed individually by a specialized pathologist with more than 25 years of experience in bone pathology, allowing assessment of whether the first, second, and third samples provided a diagnosis. The conclusion of the histopathological analysis included the results of all three samples.

### Statistical analysis

A specimen was considered diagnostic if a pathological diagnosis could be established from the biopsy sample. A single diagnostic sample was sufficient to classify the whole biopsy as diagnostic.

Different groups have been made (Fig. 1):

- Group 1: Histopathological diagnosis made on the first sample.
- Group 2: Histopathological diagnosis was not made on the first sample, and made on the second sample.
- Group 3: Histopathological diagnosis not made on the 1st or 2nd sample, made on the 3rd or subsequent sample.
- Group 4: Biopsy did not lead to a pathological diagnosis.

Odds ratios (OR) and their 95% confidence interval (CI) were computed. A univariate logistic regression model was performed to predict tumoral pathological results at the first sample, based on clinical, morphological imaging, and PET/CT parameters. Receiver operating characteristic (ROC) curves were plotted, with calculation of the area under the curve (AUC) and its confidence interval, to assess the predictive value of PET/CT quantitative parameters for tumoral pathological results at the first sample. Significance was considered for *p* < 0.05. Analyses were performed using R statistical software (R 4.3.0 GUI 1.79 Big Sur Intel build 8225 for Mac OS, The R Project^®^), along with the following packages: broom, epiDisplay, pROC, and dplyr.

A post hoc power analysis was conducted using a logistic regression model (powerLogisticCon function, *powerMediation* package in R). The calculation was based on the observed first-sample diagnostic success rate (52.6%), a total sample size of 38 patients, and a significance level of 0.05. Additionally, required sample sizes to detect odds ratios of 2 and 1.5 with 80% power were estimated under the same assumptions.

## Results

### Patient population

Patients’ characteristics are presented in Table 1. Most of the biopsies involved spinal and pelvic locations (Table 2). Metastases from breast cancer were the most common diagnosis (Table 3). Only three patients underwent a second biopsy procedure at a later date. Biopsies were performed under LA or conscious sedation (26/38). In this study, one biopsy led to a Paget result, which was considered a non-malignant biopsy. Other non-malignant biopsies resulted in bone tissue or a hemorrhagic component.

**Table 2:** Malignant biopsies’ locations

	Malignant biopsies	Non-malignant Biopsies	Tumoral biopsies *p*-value
Spine	18 (49%)	17 (61%)	
Thoracic	8 (22%)	10 (36%)	1.000
Lumbar	10 (27%)	7 25%)	0.672
Pelvic bone ring	16 (39%)	5 (18%)	0.559
Sacrum	1 (2%)	1 (3.5%)	
Iliac wing	14 (35%)	4 (14.5%)	
Pubic symphysis	1 (2%)	0 (0%)	
Upper limb	2 (5%)	1 (3.5%)	1.000
Lower limb	2 (5%)	1 (3.5%)	1.000
Skull	0 (0%)	1 (3.5%)	
Sternum	0 (0%)	3 (10.5%)	
Total	38	28

**Table 3:** Histopathologic result of malignant IGBB of the study population (*N* = 38)

Total malignant biopsy diagnoses	38
Breast cancer	21 (55%)
Bronchogenic carcinoma	4 (10%)
Plasmocytoma	4 (10%)
Urothelial carcinoma	3 (8%)
Digestive carcinoma	2 (5%)
Acute myeloid leukemia	1 (3%)
Spleen angiosarcoma	1 (3%)
Epidermoid carcinoma of the cervix	1 (3%)
B-cell lymphoma	1 (3%)

Within 66, IGBB performed, 38 were malignant diagnoses (38/66; 57.5%). Sclerotic lesions concerned 11/38 (29%) of malignant biopsies. The two most common locations were spine and pelvis (Table 2), accounting together for 34/38 (89%) of positive IGBB. Group 1 included 20 IGBB (52%), group 2 included 9 IGBB (24%), and group 3 included 9 IGBB (24%).

### Lesion-related factors

Univariate logistic regression analysis was performed to obtain odds ratios (OR) and their statistical significance. Metabolic parameters did not significantly differ between groups (Tables 4 and 5). ROC curves were generated but did not show any significant discrimination, with an area under the curve below 0.7, including 0.5 in the confidence interval (Supplementary Data 2–5). Subgroup analysis didn’t find any significant differences between groups.

**Table 4:** Association of clinical, morphological imaging, and PET/CT parameters with malignant outcome at the first sample of the biopsy

Parameters	OR	*p*-value	CI (95%)
Age	1.008	0.756	(0.959–1.059)
Sex	0.353	0.194	(0.064–1.617)
Density	0.997	0.291	(0.989–1.002)
Lesion size	1.015	0.520	(0.969–1.068)
Lytic lesion	1.166	0.825	(0.291–4.687)
Sclerotic lesion	1.114	0.880	(0.270–4.725)
Lesion SUVmax	1.079	0.341	(0.928–1.283)
Lesion SUVmean_40%_	1.118	0.407	(0.865–1.498)
Lesion MTV	1.007	0.672	(0.974–1.043)
Lesion TLG	1.002	0.463	(0.996–1.008)
Lesion SUVmax/hepatic SUVmax	1.346	0.248	(0.833–2.34)
Lesion SUVmax/vascular SUVmax	1.171	0.347	(0.854–1.686)

*OR* odds ratio, *CI* confidence interval, *MTV* metabolic tumoral volume, *TLG* total lesion glycolysis, *SUV* standardized uptake value, *IQR* interquartile range

**Table 5:** Metabolic groups parameters

Parameter	ALL	Group 1	Group 2	Group 3	Group 4
SUV_max_					
Mean	7.04	7.79	6.06	7.15	6.77
Standard deviation	5.52	4.39	2.67	5.40	7.07
Median	5.61	6.98	5.62	4.82	4.48
IQR	4.98	5.72	2.24	5.33	3.76
SUV_mean_					
Mean	4.11	4.45	3.51	4.20	4.02
Standard deviation	3.18	2.42	1.58	3.44	4.05
Median	3.22	4.15	3.19	2.91	2.73
IQR	2.89	3.40	1.19	3.32	2.2
MTV					
Mean	18.02	21.13	19.08	20.68	14.35
Standard deviation	18.55	19.45	17.07	23.55	16.92
Median	12.05	14.80	17.08	9.86	8.54
IQR	20.55	19.60	18.97	18.83	16.57
TLG					
Mean	72.17	99.4	68.11	91.03	46.16
Standard deviation	94.34	108.34	73.48	154.05	55.00
Median	34.79	40.14	55.44	28.64	25.42
IQR	73.21	141.12	54.56	59.79	48.83

*SUV* standardized uptake value, *MTV* metabolic tumor volume, *TLG* total lesion glycolysis

## Discussion

This single-center retrospective study evaluated the potential contribution of PET/CT metabolic parameters in optimizing IGBB. A total of 38 malignant bone lesions were analyzed to assess whether metabolic activity could help reduce the number of biopsy samples required for diagnosis. Despite initial hypotheses, statistical analysis did not demonstrate any significant association between PET/CT metabolic or morphological parameters (including SUVmax, SUVmean, MTV, and TLG) and biopsy diagnostic performance. Most malignant lesions were metastases (32/38), and in over half of the cases, a definitive diagnosis was obtained from the first core sample. This study addresses an underexplored topic in the literature [6], focusing on the optimization of IGBB through the integration of metabolic data from PET/CT. Biopsies were performed regardless of lesion location, whereas many studies either excluded spinal lesions or, conversely, focused on spinal location only [17, 30].

PET/CT is a hybrid imaging technique that combines CT and PET to assess tissue metabolism and identify abnormal metabolic activity. The most used radiotracer is fluorine-18 (F18) fluorodeoxyglucose (FDG—analog of glucose). The main advantages of PET/CT are early diagnosis and staging, improved diagnostic yield by revealing occult lesions and identifying viable tissue in tumors with necrosis [25]. Two necrotic lesions were excluded from statistical analyses. This is linked to increased glucose metabolism and overexpression of glucose transporter and hexokinase enzymes in tumor cells, although this varies between cancer types. FDG uptake is theoretically correlated to the amount of viable tumor cells [25]. So, a higher diagnostic yield would be attempted with biopsy targeting an area with high FDG uptake. Wang et al [31] reported an overall success rate of 84% for bone biopsies guided by PET/CT in patients with primary lymphoma. Guo et al [26] documented a 100% diagnostic success rate and sensitivity for PET/CT-guided percutaneous bone biopsies in cases of lung cancer and suspected metastatic bone disease. The results suggest that the use of PET/CT may impact the number of samples required to achieve a diagnosis.

Such discrepancies may reflect either false-positive PET/CT results or false-negative biopsies, both of which are plausible in a retrospective setting. Although multiple factors may explain this, investigating them was beyond the scope of the current study [32]. Indeed, FDG uptake can occur in inflammatory and infectious diseases, and both irradiation and immunotherapy may increase FDG uptake due to acute cellular responses to therapy in the early post-treatment phase. Conversely, certain chemotherapies may reduce FDG uptake due to a “stunning” effect. Therefore, interventional radiologists would benefit from familiarizing themselves with this imaging modality while being aware of its advantages and limitations. Furthermore, Kobayashi et al [25] highlight several benign bone tumors associated with FDG uptake, including fibrous dysplasia, giant cell tumors, Paget’s disease, non-ossifying fibromas, eosinophilic granulomas, aneurysmal bone cysts, enchondromas, and myositis ossificans. In this study, one biopsy led to a Paget result, which was considered non-malignant. They also noted that sclerotic bone metastases and some soft tissue neoplasms with low FDG uptake may be less suitable candidates for reducing the number of samples. These observations encourage caution, and radiologists should remain cognizant of the strengths and limitations of PET/CT, tailoring their approach to the clinical context. In this study, a total of 28 biopsy results were classified as non-malignant and were therefore excluded from the primary statistical analysis. These included histopathological diagnoses such as benign bone remodeling (*n* = 7), fibrosis (*n* = 6), chronic inflammatory changes (*n* = 5), bone infarct (*n* = 3), osteomyelitis (*n* = 2), Paget’s disease (*n* = 1), and nonspecific findings (*n* = 4). Their exclusion was based on methodological considerations, as the retrospective design did not allow for systematic clinical or imaging follow-up to confidently distinguish true negatives from false-negative biopsy results. Including these cases in the main analysis could have introduced diagnostic uncertainty and limited the reliability of statistical comparisons. Nonetheless, these cases remain clinically relevant, particularly regarding the interpretation of FDG-PET signals. Some of the non-malignant lesions demonstrated increased FDG uptake, highlighting the well-known overlap between neoplastic, inflammatory, infectious, and some benign bone processes with high metabolic activity. In this context, it could be of interest in future work to include such cases within a prospective framework, allowing for longitudinal clinical and imaging follow-up to confirm the benign nature of the lesions and to assess the potential for PET/CT false positives in a more robust manner.

As highlighted in the literature, other factors such as lesion density and size may be considered [4, 6, 14–16, 18, 20]. It also highlights that suspected metastases from carcinomas, which were a majority of the diagnoses, are strong indications for biopsy, given the absence of such cellular components in normal bone tissues [33, 34]. Information on sample length could have provided additional insights into biopsy adequacy [2]. Additionally, the involvement of specialized pathologists in bone pathology is described as a key factor for reliable diagnosis in this complex histopathological field.

This study has several limitations. Notably, its single-center, retrospective design introduces inherent biases, and the cohort size [35] may limit the generalizability of the findings. Moreover, potential biases are related to prior treatments. Given the retrospective nature of the study, some potentially relevant clinical data were not systematically available, including systemic treatment at the time of the PET scan and biopsy sampling, which may influence both FDG uptake and biopsy diagnostic yield. These missing variables limited the possibility of conducting a robust multivariate analysis. Furthermore, univariate statistical analysis did not reveal any significant associations between imaging or clinical parameters and the diagnostic yield. In this context, multivariate modeling was not pursued, as it would have been underpowered and of limited interpretative value in the absence of univariate signals. Even with the inclusion of selected covariates such as lesion density (in Hounsfield units) or morphological features (osteolytic, sclerotic, mixed), any multivariate model would remain exploratory and unlikely to yield additional meaningful insights. Nonetheless, future prospective studies with larger and more complete datasets could benefit from such approaches to better account for confounding factors. To further assess the robustness of our findings, a post hoc power analysis was performed. Based on our sample size (*N* = 38) and the observed first-sample diagnostic success rate (53%), the study had a power of 57% to detect an odds ratio (OR) of 2 at a significance level of 0.05. Additionally, the minimal detectable odds ratio with 80% power under the same assumptions was estimated at 2.48. These results suggest that while the study was likely underpowered to detect small-to-moderate associations, it was adequately powered to identify larger, clinically relevant effects. We also estimated the sample size required for a future prospective study. To detect an OR of 2 with 80% power and α = 0.05, a total of 66 patients would be required. Detecting a smaller effect size (OR = 1.5) would require a substantially larger cohort of 192 patients. These estimations serve as a benchmark for future studies and underscore the importance of multicenter research to better evaluate how PET/CT metrics might influence biopsy strategies. In addition, the interval between PET/CT and biopsy-up to 90 days-may have introduced variability in FDG uptake due to possible disease progression or treatment effects. However, this 3-month delay was intentionally chosen as a pragmatic compromise, in line with routine clinical workflows, and to ensure inclusion of a sufficiently representative cohort. Although longer delays may affect metabolic activity, we considered this timeframe to be clinically acceptable. Moreover, we did not systematically document treatment status at the time of PET/CT or biopsy, which is an acknowledged limitation. That said, in most cases, biopsies were performed as part of the initial diagnostic workup, before the initiation of systemic therapy. Therefore, the majority of patients were likely treatment-naïve at the time of both PET/CT and biopsy. Some biopsies (7/38, 18%) were performed after the initiation of radiotherapy or chemotherapy. However, as this variable was distributed evenly across groups, it does not appear to be a significant confounding factor. That said, in most cases, biopsies were performed as part of the initial diagnostic workup, before the initiation of systemic therapy. Therefore, the majority of patients were likely treatment-naïve at the time of both PET/CT and biopsy. In addition, use of a drill system, commonly employed for sclerotic lesions (12/38 patients; 32%) [36], can introduce crush artifacts that complicate histological analysis [1]. Concerning needles, 11- and 13-gauge needles were used, which did not differ between groups. Although larger-gauge needles are theoretically associated with improved sample quality, evidence in the literature remains inconclusive [2, 9, 13]. Another limitation concerns the lack of a standardized evaluation of biopsy sample quality. Data on core length, fragmentation, or tissue cellularity were not available, due to the retrospective nature of the study and the absence of a systematic documentation of these parameters in pathology reports. Moreover, retrospective evaluation of histological quality is inherently subjective and influenced by multiple interdependent factors—including tissue architecture, preservation, and diagnostic interpretability—which are not always explicitly described. Therefore, it was not possible to determine whether certain samples (e.g., the third core) were of superior quality or contributed more to the final diagnosis. This limits our ability to fully distinguish between sampling adequacy and potential metabolic correlation.

PET/CT data analysis underscores the importance for interventional radiologists to remain cautious of potential false-positive findings, including non-malignant FDG uptake and imaging artifacts. For further research, prospective studies with larger cohorts are needed, as well as a standardized protocol for PET/CT data.

The findings of the present study suggest that taking fewer than three biopsy samples may compromise diagnostic accuracy and that the amount of tissue obtained for histopathological evaluation remains crucial, even in cases of high PET-scan uptake. Moreover, biopsy site selection should not be solely based on the most metabolically active lesion, as each clinical scenario presents distinct challenges for the interventional radiologist. While minimal sampling helps to streamline procedures, it remains essential to ensure the quantity of the material collected. Striking a balance between procedural efficiency and diagnostic reliability is key to optimizing bone biopsy practice.

## Conclusion

This study addresses an underexplored topic in the literature and provides relevant findings regarding the consideration of lesion metabolism in the performance of IGBB. Current recommendations suggest obtaining at least three biopsy samples and emphasize the importance of selecting a site with the lowest procedural risk. Our results indicate that, even in highly metabolic lesions, PET/CT alone does not allow for a reduction in the number of samples without compromising diagnostic performance. However, PET/CT remains the tool of choice for assessing disease extent and is a valuable aid in biopsy site selection. This has significant implications for musculoskeletal interventional radiologists in daily practice.

## Supplementary information

ELECTRONIC SUPPLEMENTARY MATERIAL

## Abbreviations

FDG: FluoroDeoxyGlucose
IGBB: Image-guided bone biopsy
IQR: Interquartile range
LA: Local anesthesia
MTV: Metabolic tumor volume
OR: Odd ratio
PET/CT: Positron emission tomography/computed tomography
ROC: Receiver operating characteristic
SUV: Standardized uptake value
TLG: Total lesion glycolysis
