# Supplementary material for: Performance of image-guided bone biopsies in malignant lesions: impact of PET/CT metabolic activity on the number of samples required
Source: Insights Imaging. 2025 Oct 31;16:236. doi: 10.1186/s13244-025-02130-2 (PMC12579014; doi:10.1186/s13244-025-02130-2)
Supplement: Supplementary file 1 — ELECTRONIC SUPPLEMENTARY MATERIAL [file 13244_2025_2130_MOESM1_ESM.pdf]

Performance of Image-Guided Bone Biopsies in Malignant Lesions: Impact of PET/CT Metabolic Activity on the Number of Samples Required

ELECTRONIC SUPPLEMENTARY MATERIAL

SUV (Standardized Uptake Value)	Commonly used index to assess the FDG uptake. It is defined as the concentration of tracer activity in a volume of interest divided by the injected dose per unit of body weight
SUV_{max}	Most use data, corresponds to the maximum metabolic activity of one voxel with the volume of interest (VOI). Non-operator dependent but depends on imaging protocol
SUV_{mean}	Mean value in the volume of interest. Operator dependent
Metabolic Tumor Volume (MTV) - $SUV_{40\%}$	Corresponds to functional tumoral volume<br>$SUV_{40\%}$ - Automatic threshold, expressed as a percentage of the maximum SUV value in the delimited volume of interest. Minimal user dependency
Total Lesion Glycolysis (TLG)	Product of lesion SUVmean and metabolic tumor volume

SUPPLEMENTARY DATA 1. Metabolic characteristics of interest

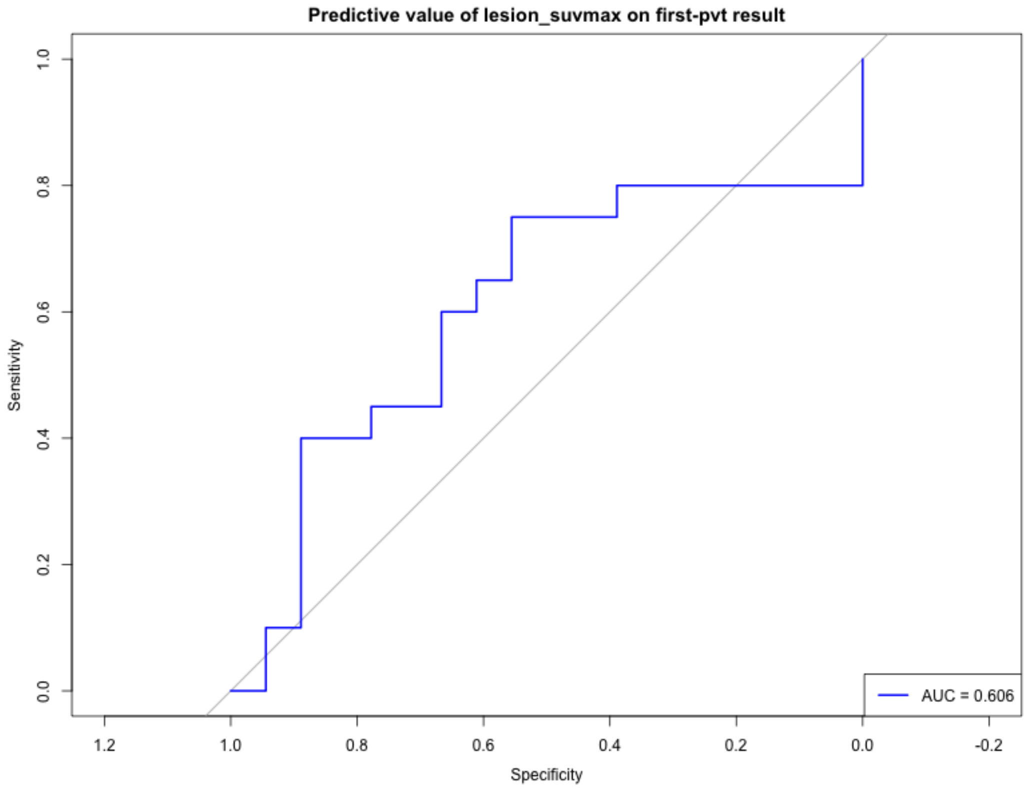

SUPPLEMENTARY DATA 2. ROC curve of SUV_{max} data

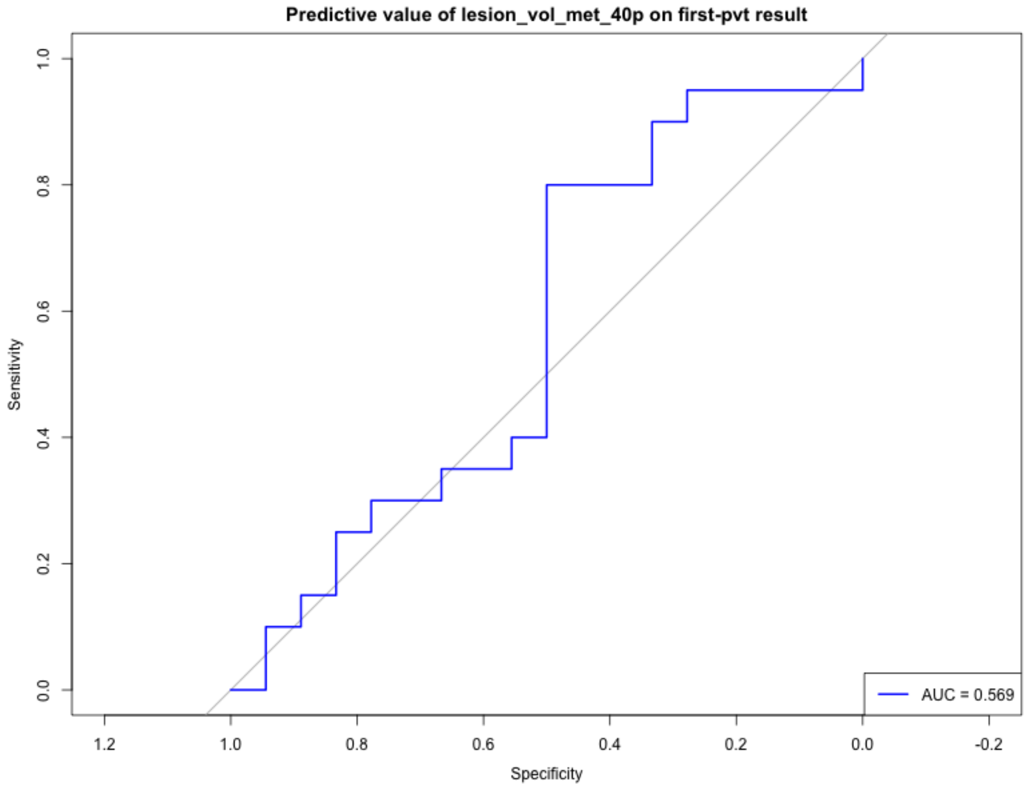

SUPPLEMENTARY DATA 3. ROC curve of VTM data

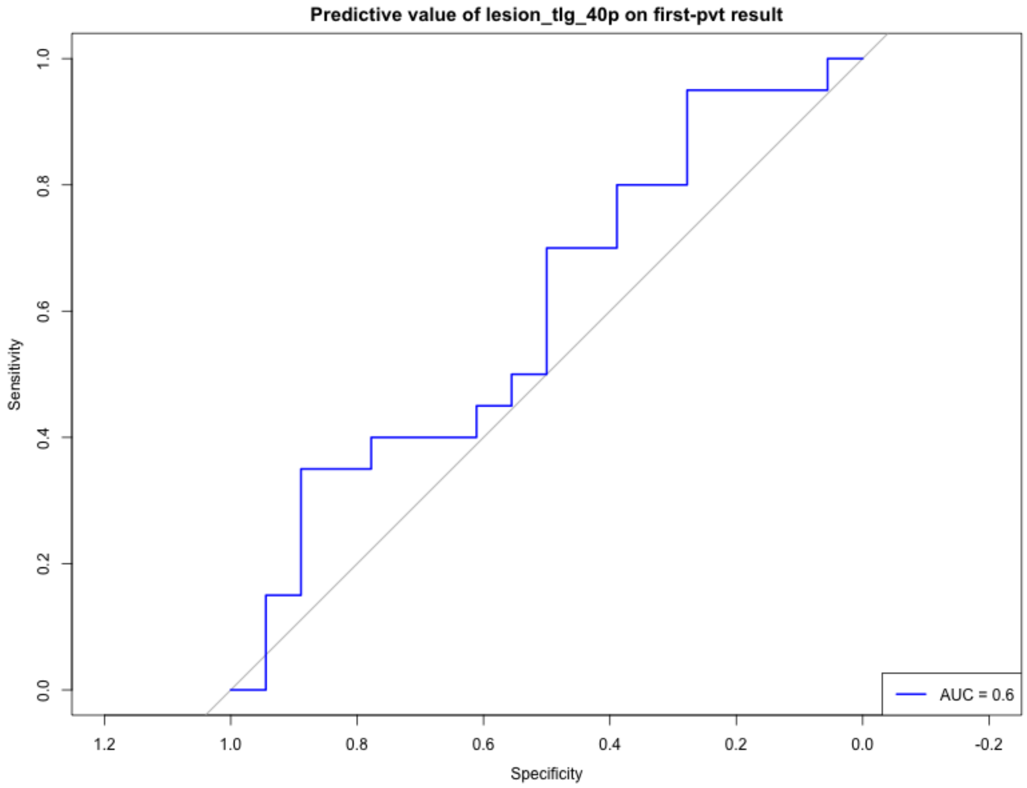

SUPPLEMENTARY DATA 4. ROC curve of TLG data

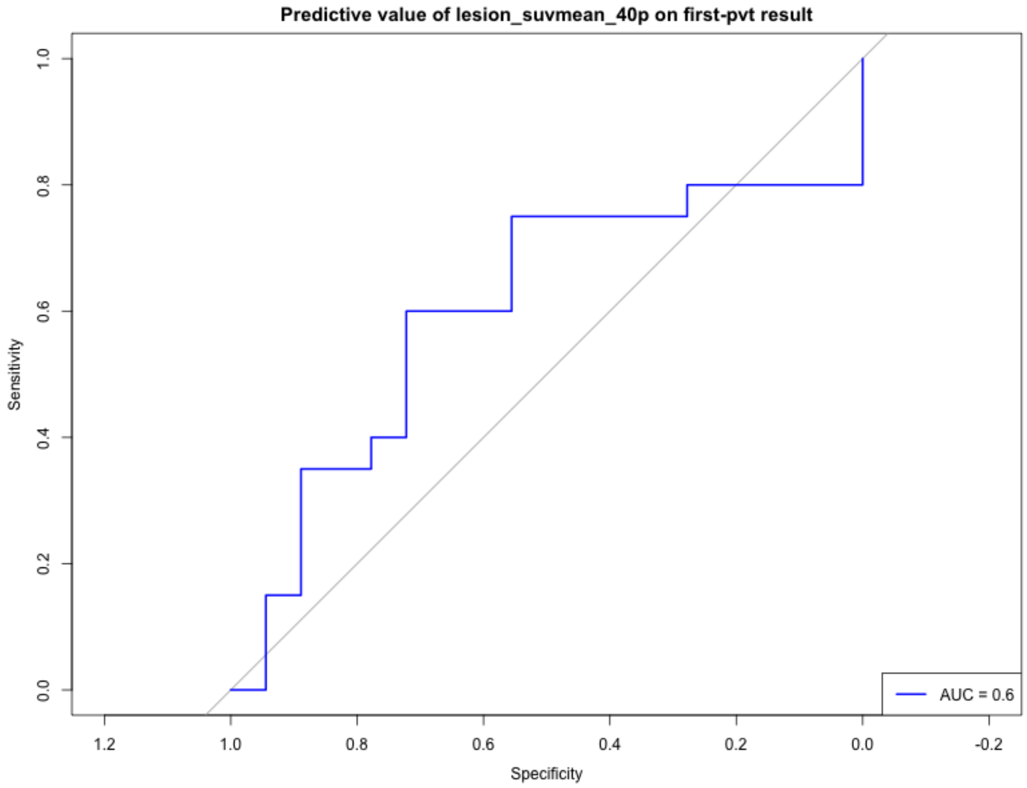

SUPPLEMENTARY DATA 5. ROC curve of SUV_{mean} data
